# Perceived cognitive impairment and occupational functioning in prostate cancer survivors: an exploratory cross-sectional analysis

**DOI:** 10.1007/s11764-025-01743-2

**Published:** 2025-01-15

**Authors:** Lorna Pembroke, Kerry A. Sherman, Haryana M. Dhillon, Heather Francis, David Gillatt, Howard Gurney

**Affiliations:** 1 Lifespan Health and Wellbeing Research Centre, Macquarie University, Sydney, NSW 2109 Australia; 2 Faculty of Medicine, Health and Human Sciences, School of Psychological Sciences, Macquarie University & Macquarie University Hospital, Sydney, NSW 2109 Australia; 3 Centre for Medical Psychology and Evidence-Based Decision-Making, Faculty of Science, School of Psychology, University of Sydney, Sydney, NSW 2006 Australia; 4 Psycho-Oncology Cooperative Research Group, Faculty of Science, School of Psychology, University of Sydney, Sydney, NSW 2006 Australia; 5 Macquarie University Urology Clinic, Faculty of Medicine and Health Sciences, Macquarie University & Macquarie University Hospital, Sydney, NSW 2109 Australia; 6 Macquarie University Clinical Trials Unit (CTU), Faculty of Medicine and Health Sciences, Macquarie University & Macquarie University Hospital, Sydney, NSW 2109 Australia

**Keywords:** Return to work, Occupational functioning, Cognitive functions, Cancer-related cognitive impairment, Rehabilitation, Prostate cancer

## Abstract

**Purpose:**

Perceived cancer-related cognitive impairment (CRCI) has been reported in prostate cancer survivors. Little is known about how CRCI impacts occupational functioning in working-aged prostate cancer survivors (PCS). This study aimed to investigate the association between CRCI and occupational functioning in PCS.

**Methods:**

Data from 51 PCS, who were employed at the time of diagnosis, undergoing hormonal treatments (e.g., androgen deprivation therapy) or ‘watchful waiting’/ ‘active surveillance’, were analysed. An online survey assessed CRCI using the FACT-Cog Perceived Cognitive Impairments (PCI20) subscale, the EORTC-QLQ-30 two-item cognitive functioning scale, and a single ‘Yes/No’ CRCI item (i.e., were ‘changes in thinking (e.g., memory, attention)’ experienced as a treatment side effect). PCS also indicated ‘Yes/No’ to changes to their ability to work, performance of work duties, and decreased work hours. Logistic regression analyses examined the relationship between CRCI measures and occupational outcomes.

**Results:**

Of the 51 PCS, 19 (37%) endorsed experiencing cognitive side effects from treatment. The single ‘Yes/No’ CRCI question was significantly associated with perceived changes in work ability and ability to perform work duties at the same level. PCI20 and the EORTC-QLQ-30 cognitive functioning scale were not significantly associated with any occupational outcomes.

**Conclusion:**

Perceived CRCI is associated with adverse changes to occupational functioning and is important to consider when PCS are making plans to return-to-work following treatment.

**Implications for Cancer Survivors:**

Prostate cancer survivors may experience cognitive changes, which may impact their work ability.

**Supplementary Information:**

The online version contains supplementary material available at 10.1007/s11764-025-01743-2.

## Introduction

Prostate cancer is one of the most prevalent malignancies affecting biological males worldwide. Though most diagnoses occur around age 65 years or older, advancements in screening and treatment have led to earlier detection and relatively high survival rates (e.g., > 93% 5-year survival) [1]. There is an increasing population diagnosed with prostate cancer while of working age, especially as retirement age increases [2]. However, the diagnosis and treatment side effects can impact occupational functioning or ‘work ability’, the ability to perform roles and responsibilities associated with one’s occupation effectively [3, 4]. Moreover, this can lead to early retirement plans, potentially reducing financial security and threatening self-identity [4].

Employment can positively impact both mental and physical well-being and be integral to the recovery process and resuming normal life, leading to improved quality of life and self-esteem in prostate cancer survivors (PCS) [5]. In PCS, employment has also been demonstrated to be crucial for one’s sense of masculinity, autonomy, and financial stability [2, 6]. However, clinicians and cancer survivors often underestimate the challenges of returning to work [6]. High rates of work absenteeism, changes to work expectations, and diminished capacity following prostate cancer treatment, including prostatectomy, radiation therapy, hormonal treatments, and combined modalities, have been reported in several studies [6–8]. Moreover, PCS are more likely to retire earlier than planned and receive early retirement pensions [9, 10]. While this finding may be associated to diagnoses at an older age, the risk of early retirement was over fourfold compared to non-cancer controls and also exceeded other cancer types including non-Hodgkin’s, colorectum, breast, kidney, bladder, cervix, testes, and melanoma cancers, even after adjusting for age [10]. As PCS are at a higher risk of early retirement, identifying and addressing factors affecting occupational functioning is important for enhancing survivorship.

A range of factors associated with disruption to occupational functioning in PCS have been identified including treatment side effects, older age, advanced cancer stage, surgery/radical prostatectomy, chemotherapy, medical comorbidities, low education and socioeconomic status, weak support/negative attitudes at work, and poor emotional functioning [11]. Physical side effects of prostate cancer treatment, including fatigue, muscle loss, urinary incontinence, and bowel and bladder problems, are more commonly reported in the literature as challenging factors associated with return to work [12–16]. Little is known, however, about the impact of cancer-related cognitive impairment (CRCI) on occupational functioning.

Cognitive problems following prostate cancer treatments, such as androgen deprivation therapy (ADT), are experienced reportedly by 10 to 69% of PCS [17]. Several prospective studies have identified declines in processing speed, attention, language skills, memory, visuospatial functioning, and executive functioning on neuropsychological testing [18, 19], with PCS on ADT performing worse than non-cancer controls [20–22]. Though advantages of comprehensive neuropsychological assessments include robust construct validity, bias reduction, detection of subtle cognitive changes, and ability to make normative comparisons, these assessments are not always feasible to administer due to limited access to specialised professionals, financial constraints, and participants’ time burden [23]. Moreover, in this testing, cognition is assessed in optimal conditions (one-to-one distraction-free) and may not always reflect reported day-to-day challenges [23]. In contrast, self-report cognitive measures provide an efficient, accessible, economical alternative for gathering information on the functional impact of perceived CRCI [23]. Being closely associated with quality of life, psychological distress, and everyday functioning [23–25], subjective reports can serve as a screener highlighting potential future intervention and as an indicator of future cognitive decline [26]. Bearing in mind that concerns around the validity of subjective assessments of ‘cognition’ include its susceptibility to response bias, fluctuations in perceived cognition or mood, and the level of one’s insight [23].

Cognitive difficulties in PCS may interfere with occupational capacity and performance, with significant implications for social and personal responsibilities, quality of life, finances, and mental health [27]. In a mixed cancer sample, 62% of respondents in a survey reported disruptive impacts of CRCI on occupational and domestic tasks and relationships [28]. Difficulties with attention/concentration, processing speed, memory/learning, and executive functioning have been identified by cancer survivors as highly disruptive to work-related outcomes, including work performance, productivity, and perceived occupational ability [29–35]. Perceived cognitive impairment was also a predictor of return-to-work outcomes, along with treatment, age, perceived work ability, and mental work ability in mixed cancer samples that included breast, haematological, genitourological, and gastrointestinal cancers [36]. Moreover, CRCI-related disruptions to self-confidence and self-esteem have also been observed with some cancer survivors, for example, reporting social withdrawal due to embarrassment related to memory difficulties [32]. In PCS, more specifically, little is known about the impact of CRCI on work-related outcomes.

Widely used self-report measures of perceived cognitive functioning in cancer populations, such as the Functional Assessment of Cancer Therapy-Cognitive Subscale (FACT-Cog) [37] and the cognitive functioning scale from the European Organisation for Research and Treatment of Cancer Quality of Life Questionnaire (EORTC-CF) [38], often rely on assessors to score patient responses and compare their responses with norms. Given that clinical appointments are often constrained by time [39], a brief screener of CRCI in PCS could be beneficial to rapidly identify PCS who may benefit from more detailed CRCI assessment and interventions treating or managing CRCI [40]. Brief screening measures that are easily administered, interpretable, inexpensive, and efficient have greater success and usefulness in being incorporated into routine clinical assessments [41]. Discrete, binary ‘Yes/No’ measures may be particularly appropriate for settings with culturally and linguistically diverse clientele or lower levels of health literacy [40]. Moreover, exploring cognitive changes over a broader timeframe, such as since cancer diagnosis/treatment, rather than over the past week, as utilised by the FACT-Cog and EORTC-CF, may provide helpful insights into the perceived progression and cumulative impact of a condition. Overall, limited research exists on the identification of PCS concerns using a ‘Yes/No’ question of CRCI.

This exploratory study aimed to investigate the association between perceived CRCI and occupational functioning in PCS. Several approaches to assessing CRCI were used including a set of simple ‘Yes/No’ questions regarding CRCI and work capacity, as well as the Perceived Cognitive Impairments (PCI) subscale from the FACT-Cog [37] and the EORTC-CF [38] questionnaires. Documenting possible associations between CRCI and work in this population may help inform targeted interventions, such as psychotherapy, cognitive rehabilitation, or cognitive behaviour therapy, to support return-to-work plans for PCS.

## Methods

### Design

This study reports a subset of data on occupational functioning collected within a larger mixed-methods cross-sectional survey investigating perceived cognitive functioning in PCS and its relationship with various psychosocial, biomedical, demographic, and lifestyle factors. The cross-sectional survey was registered with the Australian New Zealand Clinical Trials Registry (25 November 2021; registration number: ACTRN12621001608853). Self-report questionnaires were administered online using Qualtrics software (Provo, UT) and REDCap (Research Electronic Data Capture) [42] or via paper copies distributed in clinic waiting rooms. The Strengthening the Reporting of Observational Studies in Epidemiology (STROBE) checklist was used to guide the reporting of this study [43]. Ethical approval was obtained from the Macquarie University Human Research Ethics Committee (reference number: 52020611919011).

### Participants

Adult English-literate PCS, who were either undergoing hormonal treatments (e.g., androgen deprivation therapy) or on ‘watchful waiting’/ ‘active surveillance’, were recruited from Australian healthcare professionals, the HealthMatch research register, social media (i.e., Facebook, Twitter), community groups (e.g., Men’s Sheds, Probus Clubs), and by word of mouth. Participants were recruited between January 2021 and October 2023. As this study was exploratory in nature, we included participants with prostate cancer at all stages and with various treatment histories, excluding those who began treatment after retirement.

### Materials

Demographic, prostate cancer-related, and medical details were obtained. There were three measures of perceived cognitive functioning: (1) the Perceived Cognitive Impairments–20 items (PCI20) subscale from the FACT-cog [37, 44, 45], (2) the EORTC-CF [38], and (3) a single ‘Yes/No’ item to whether ‘changes in thinking (e.g., memory, attention)’ were experienced as a treatment side effect. The PCI20 assesses difficulties with attention, processing, memory, and language through the rating of the frequency of cognitive complaints over the week on a 5-point scale, from 0 = ‘never’/ ‘not at all’ to 4 = ‘several times a day’/ ‘very much’. Higher scores represent better cognitive function (range = 0–80) with a cutoff of ≤ 59.5 indicating perceived low cognitive functioning (78.8% sensitivity, 84.1% specificity) in mixed cancer samples including those diagnosed with breast, colorectal, gynaecological, lunch, genitourinary, and prostate cancers [40]. Cronbach’s alpha was 0.97 in this study.

EORTC-CF entails two items assessing attention and memory: (1) ‘Have you had difficulty in concentrating on things, like reading a newspaper or watching television?’ and (2) ‘Have you had difficulty remembering things?’ [38]. These items are rated on a 4-point scale (‘Not at all’ to ‘Very much’) to form a total score (0 to 100) with higher scores signifying better cognitive functioning (ref). A cutoff of ≤ 75.0 indicates perceived low cognitive functioning (90.9% sensitivity, 57.1% specificity) in mixed cancer samples [40]. Cronbach’s alpha for this scale was 0.73 in this study.

A single ‘Yes/No’ item of whether ‘changes in thinking (e.g., memory, attention)’ were experienced as a treatment side effect was developed as an exploratory question due to its straightforward, discrete binary response, eliminating the need for a cutoff score.

Occupational functioning following cancer diagnosis and treatment was assessed with three ‘Yes/No’ questions developed by the researchers: (1) ‘After commencing treatment for your prostate cancer, did you notice any changes to your ability to work?’; (2) ‘Have you been able to perform your work duties at the same level as before?’; (3) ‘Have you decreased your hours at work?’. These researcher-developed items have not undergone validation in any cancer populations and were included in the primary cross-sectional survey for exploratory purposes.

### Statistical analysis

The sample size was determined by the available data, that is, only survey participants completing the demographic/medical questions, along with PCI20 were included for analysis. Statistical analyses were performed using Statistical Package for the Social Sciences (SPSS, version 29). A significance level of *α* = 0.05 was employed for all statistical analyses. To identify bivariate associations and potential covariates for later logistic regression analyses, one-way analysis of variance (ANOVA; for continuous variables) or Fisher’s exact tests (for categorical variables) were conducted between each demographic/medical variable and the three ‘Yes/No’ occupational functioning outcomes. The association between the three measures of perceived cognitive functioning was analysed using ANOVAs (with the Yes/No question of CRCI as the independent variable, and PCI20 and EORTC-CF each as dependent variables) and Pearson’s correlation analysis (between PCI20 and EORTC-CF).

To assess the association of each measure of perceived cognitive functioning with the three occupational functioning outcomes, three sets of three logistic regression analyses were performed including any identified covariates. For the PCI20 and EORTC-CF, both the continuous variable and cutoffs for low cognitive functions were analysed separately as predictors for the logistic regression analyses. The fit of the logistic regression model was assessed using Hosmer–Lemeshow tests. Cases with missing data were excluded from specific analyses to ensure result integrity and validity, employing listwise deletion.

## Results

Table 1 displays the descriptive demographic, prostate cancer-related, and medical data of the sample. Additional details are outlined in Tables S1 to S2 in the Supplementary Materials. From a total of 96 PCS recruited, 51 were included for analysis who did not retire prior to receiving prostate cancer treatment. Participants were aged between 54 and 85 years (*M* = 69.9, *SD* = 7.2) with most having completed tertiary education (57%), were currently retired (61%), had early/localised/regional prostate cancer (55%), and were undergoing hormone therapy (73%).

**Table 1:** Demographic, prostate cancer-related, medical characteristics of the sample

	*M* (SD), *N* (%), range
*Demographic*	
Age (*n* = 51)	*M* = 69.9 (*SD* = 7.2), range = 54.4–84.6
Relationship	
Partnered	43 (84%)
Not partnered	8 (16%)
Education level	
Did not complete year 12	8 (16%)
Year 12/vocational training	14 (28%)
Undergraduate/postgraduate	29 (57%)
Current employment status	
Full-time/part-time/looking for employment/self-employed/carer	20 (39%)
Retired	31 (61%)
Self-reported employment type	
Clerical and sales/service/agricultural/machines trade/structural work, e.g., construction	14 (27%)
Professional, technical, and managerial occupations	30 (59%)
Other	7 (14%)
No. of children (*n* = 44)	*M* = 2.5 (*SD* = 1.0)
Children living at home	
Yes	6 (12%)
No	38 (75%)
Country of birth	
Aus/NZ	32 (63%)
US/UK/Europe	13 (26%)
Asia/South America/Africa	6 (12%)
Location of residence	
Metropolitan	36 (71%)
Regional	14 (28%)
Languages spoken other than English	
No	42 (82%)
Yes	8 (16%)
*Prostate cancer details*	
Stage	
Early/regional	28 (55%)
Metastatic	23 (45%)
Time since diagnosis in years (*n* = 50)	*M* = 6.2 (*SD* = 5.5), range = 0.25–22.2
Cancer treatments	
Watchful waiting/active surveillance	17 (33%)
Surgery	18 (35%)
Hormone therapy (e.g., ADT)	37 (73%)
Radiation therapy	25 (49%)
Chemotherapy	6 (12%)
Immunotherapy	3 (6%)
*Medical details*	
Number of non-cancer medications (*n* = 42)	*M* = 3.5 (*SD* = 3.2), range = 0–15
No. of comorbidities* (*n* = 50)	*M* = 0.8 (*SD* = 0.7), range = 0–2
Mental health diagnosis (*n* = 51)	11 (22%)

*This included heart condition, diabetes, hypertension, neurological conditions, stroke, seizures, and head injury

The bivariate associations of each demographic, prostate cancer-related, and medical variables with each of the three occupational outcomes are outlined in Table S3 in the Supplementary Materials. ‘Changes to work ability’ was significantly associated with hormone therapy (*p* = 0.007), chemotherapy (*p* = 0.009)*,* and the number of medical comorbidities (*F*(1, 45) = 7.41, *p* = 0.009). No significant bivariate associations were identified with ‘ability to perform work duties at the same level’. ‘Decreased work hours’ was significantly associated with hormone therapy (*p* = 0.049). These significant variables were incorporated as covariates in relevant logistic regression analyses exploring the relationship between perceived cognitive functioning and occupational functioning.

On the Yes/No item, 19 (37%) out of 51 PCS endorsed experiencing cognitive side effects. The mean PCI20 score was 59.2 (*SD* = 17.8, *n* = 51) with 41% of PCS meeting cutoff for low perceived cognitive functioning. The mean EORTC-CF score was 80.3 (*SD* = 20.4, *n* = 50) with 35% of PCS meeting cutoff for low perceived cognitive functioning. Regarding the associations between these subjective cognitive measures, the mean PCI20 score for those endorsing cognitive side effects (*M* = 50.9, *SD* = 14.1) was significantly lower non-endorsers (*M* = 64.1, *SD* = 18.12), *F*(1,49) = 7.41, *p* = 0.009. Similarly, the mean EORTC-CF score of PCS endorsing cognitive side effects (*M* = 71.3, *SD* = 20.5) was also significantly lower than non-endorsers (*M* = 85.4, *SD* = 18.8), *F*(1,48) = 6.10, *p* = 0.017. PCI20 and EORTC-CF (*r* = 0.50, *p* < 0.001) were positively associated.

### Perceived cognitive functioning and its association with occupational functioning

As depicted in Table 2, a ‘Yes/No’ question of cognitive side effects was significantly associated with changes of (1) work ability and (2) ability to perform work duties at the same level after controlling for relevant demographic, prostate cancer-related, or medical covariates. This question was not significantly associated with decreased work hours when controlling for hormone therapy.

**Table 2:** Logistic regression analyses of changes to occupational functioning questions with cognitive changes controlling for covariates

	*β*	*SE β*	Wald’s	*df*	*p* value	e^β^(95% C.I.)
*(1) Changes to your ability to work?*						
Cognitive changes	1.64	0.79	4.32	1	0.038	5.17 (1.10–24.34)
Hormone therapy	1.71	1.17	2.13	1	0.144	5.51 (0.56–54.51)
Chemotherapy	0.84	1.25	0.45	1	0.501	2.32 (0.20–26.91)
Medical comorbidities	− 0.93	0.56	2.79	1	0.095	0.39 (0.13–1.18)
Constant	− 1.87	1.15	2.64	1	0.104	
*(2) Ability to perform work duties at the same level?*						
Cognitive changes	− 1.24	0.62	4.06	1	0.044	0.289 (0.09–0.97)
Constant	0.79	0.38	4.27	1	0.039	
*(3) Decreased work hours?*						
Cognitive changes	1.14	0.70	2.67	1	0.102	3.14 (0.80–12.40)
Hormone therapy	1.23	0.72	2.87	1	0.09	3.41 (0.83–14.06)
Constant	− 1.01	0.63	2.59	1	0.107	

Similar logistic regression analyses with the PCI20 or EORTC-CF as a predictor variable (either as a continuous variable or dichotomous, based on clinical cutoff scores) were conducted. These regression models yielded no significant associations with any of the occupational outcomes (see Supplementary Material 5 and 7). Additionally, PCS endorsing changes to work ability, not being able to perform work duties at the same level, and experiencing decreased work hours, had lower mean scores on the PCI20 and EORTC-CF than those who did not endorse these occupational items, although these findings were not statistically significant (see Tables S6 and S8 in the Supplementary Material).

## Discussion

This study explored the association between perceived cognitive impairment and occupational functioning in prostate cancer survivors (PCS). More than a third of PCS endorsed experienced cognitive changes or reported low cognitive function. Preliminary data on a single ‘Yes/No’ question of CRCI yielded significant associations with work ability, which was not demonstrated using the EORTC-CF and PIC20. Prostate cancer treatment type and medical comorbidities were also associated with occupational outcomes.

Though perceived cognitive changes following prostate cancer treatment are not uncommon, its implications are not recognised widely in survivorship care [17]. Our findings were consistent with prior cancer survivor research documenting the link between CRCI and changes to work capacity [27, 46]. CRCI has been regarded as a distressing issue and a perceived barrier to being promoted, appointed to important projects, or returning to pre-cancer levels of work [29–34, 47]. In PCS more specifically, cognitive declines in attention/concentration, processing speed, language skills, memory, visuospatial functioning, and executive functioning have been documented [18–22]. These cognitive domains, along with fatigue, have been identified as the most problematic for occupational functioning in cancer survivors [29–35]. Cognitive difficulties may influence beliefs about work expectations and work-related self-efficacy, one’s confidence in their ability to perform job tasks [34, 48, 49]. Subjective appraisals of work abilities have been found to strongly predict return to work in cancer patients regardless of age, diagnosis, fatigue, quality of life, or psychological or physical complaints, reflecting how underlying perceptions of illness may shape coping strategies and functional outcomes [36]. PCS-specific research in this area, however, is sparse and requires further investigation. Since subjective and objective cognitive functioning may not always align [23–25], their impact on occupational functioning may also differ. Additional research is thus needed to clarify the impact of perceived and objective CRCI and the role of screening, assessment, and treatment on work-related outcomes.

Several reasons may explain the disparate associations between the measures of cognitive functions and work ability. The ‘Yes/No’ question specifically examined cognitive changes since cancer diagnosis and treatment, whereas the EORTC and PCI20 examined perceived cognitive functioning over the past week. Though the EORTC and PCI20 may be more sensitive to change over time, low cognitive functioning identified on the EORTC and PCI20 may possibly be perceived as being attributed to factors other than cancer, such as life stressors, general ageing, or fluctuations in mood. Whereas a ‘Yes/No’ question of CRCI may overly simplify a complex, multifaceted phenomenon like cognitive functioning, it may be subjected to greater individual variability in interpretation due to its limited ability to capture a nuanced range of responses reflecting varying degrees of perceived impairment. Nevertheless, there may be potential to implement a stepped care model. Survivors endorsing problems on the ‘Yes/No’ question CRCI may be led to opportunities for further assessment of CRCI and its impact, such as completing a longer questionnaire, = like the FACT-Cog. Depending on the outcome of further assessment, it may flag the need for a comprehensive neuropsychological assessment and/or prompt clinical monitoring and intervention, such as cognitive rehabilitation. Further research, however, is required to assess the utility of a single ‘Yes/No’ question of CRCI, ascertaining its test–retest reliability, predictive validity, concurrent validity, and other psychometric properties.

Consistent with previous literature on factors associated with adverse work outcomes in cancer survivors [50], we found the number of comorbidities and cancer treatment types was associated with perceptions of work ability and decreased work hours. More specifically, hormone therapy and chemotherapy treatments for prostate cancer likely reflect more advanced cancers whereby ongoing or long-term physical side effects, including pain, fatigue, and urinary incontinence, most likely limit occupational participation and impact work-related decisions [2]. This may also explain in part why CRCI was no longer associated with decreased work hours, when hormone therapy was controlled for in the model.

This cross-sectional study with a relatively small sample size has several limitations. These limitations include the inability to disentangle the contribution of other symptoms and factors apart from CRCI on work capacity. For instance, fatigue, physical side effects (e.g., urinary problems), and psychological distress, such as anxiety and depression, also hinder occupational functioning, delay return-to-work, and are associated with semi-retirement/retirement from work [51, 52]. Other factors contributing to employment-related decisions include the nature of the work itself (e.g., level of physical/cognitive demand, risk-associated, job stress, support, and flexibility) [53, 54] and legal/societal policies (e.g., access to disability pension) [8]. Moreover, cancer itself may elicit a re-evaluation of priorities between career aspirations, health, and interpersonal relationships, whereby work-related pursuits hinder efforts to the latter priorities [4, 6]. In addition, the impact of CRCI may vary in relation to treatments administered and dosage [55]. Work-related outcomes investigated by this study were limited as they did not take into consideration sick leave status or the variability in sick leave accessible to different individuals. Self-reported measures may be subjected to biases related to awareness (i.e., potentially overestimating or underestimating one’s abilities) [25]. Furthermore, the nature and magnitude of specific cognitive domains (e.g., memory, attention, executive functioning) affected and their contribution to disruptions in work-related outcomes could not be determined, underscoring the need for future research to address this gap with the incorporation of non-cancer controls.

Future research should further explore the nature and impact of CRCI-related occupational challenges on broader aspects of quality of life, including social functioning, work-related self-efficacy, and financial planning to inform assessment and target interventions to support return to work for PCS. Though evidence supporting the delivery of neuropsychological intervention, such as cognitive rehabilitation for cancer survivors, is gaining more attention [56], its effectiveness in PCS has not been determined, especially in the context of work-related outcomes. Nevertheless, research supports education about prostate cancer, its treatments, and potential side effects, along with individual discussions, as a means of enhancing work outcomes and psychological wellbeing [57]. Though poor self-perceptions of work capability have been found to be associated with postponed engagement with employment in PCS [58], future studies should also consider using performance-based measures.

In conclusion, our findings indicate that CRCI may interfere with occupational functioning in PCS. Screening for, assessing, and treating CRCI and CRCI-related work issues should be comprehensively explored as part of return-to-work planning. A binary, single-item screening measure for CRCI may be clinically valuable as an initial step in identifying potential work-related CRCI issues in PCS.

## Supplementary Information

Below is the link to the electronic supplementary material.

Supplementary file1 (DOCX 976 KB)

## Data Availability

Data and materials may be made available upon request.
