# Supplementary material for: Perceived cognitive impairment and occupational functioning in prostate cancer survivors: an exploratory cross-sectional analysis
Source: J Cancer Surviv. 2025 Jan 15;20(4):1412–20. doi: 10.1007/s11764-025-01743-2 (PMC13375849; doi:10.1007/s11764-025-01743-2)
Supplement: Supplementary file 1 — Supplementary file1 (DOCX 976 KB) [file 11764_2025_1743_MOESM1_ESM.docx]

# Supplementary Material

[Supplementary Material 1](#_Toc181356293)

[Table S1. Additional prostate cancer details 2](#_Toc181356294)

[Table S2. Medical comorbidities details 2](#_Toc181356295)

[Table S3. Covariate Analysis with *p-*values 3](#_Toc181356296)

[S5: SPSS output of logistic regression analysis using PCI20 as a predictor of occupational outcomes 5](#_Toc181356297)

[(1) Changes to your ability to work? 5](#_Toc181356298)

[(2) Ability to perform work duties at the same level? 6](#_Toc181356299)

[(3) Decreased work hours? 6](#_Toc181356300)

[Table S6. PCI20 Means and Standard Deviations for Occupational Outcomes 8](#_Toc181356301)

[S7: SPSS output of logistic regression analysis using EORTC-QLQ CF as a predictor of occupational outcomes 9](#_Toc181356302)

[(1) Changes to your ability to work? 9](#_Toc181356303)

[(2) Ability to perform work duties at the same level? 9](#_Toc181356304)

[(3) Decreased work hours? 10](#_Toc181356305)

[Table S8. EORTC-CF Means and Standard Deviations for Occupational Outcomes 11](#_Toc181356306)

## Table S1. Additional prostate cancer details

|  | **M (SD), N (%), range** |  |  |  |
| --- | --- | --- | --- | --- |
| Surgery type |  |  |  |  |
| - Radical prostatectomy | 15 (31%) |  |  |  |
| - Other | 2 (4%) |  |  |  |
| Hormonal treatment |  |  |  |  |
| - Goserelin | 17 (33%) |  |  |  |
| - Leuprolide | 8 (16%) |  |  |  |
| - Bicalutamide | 6 (12%) |  |  |  |
| - Enzalutamide | 3 (6%) |  |  |  |
| - Leuprorelin | 4 (8%) |  |  |  |
| - Abiraterone | 6 (12%) |  |  |  |
| - Other | 3 (6%) |  |  |  |
| Discontinuation of HT due to side-effects previously | 5 (10%) |  |  |  |
| HT administration route |  |  |  |  |
| - Monthly injections | 3 (6%) |  |  |  |
| - Four-monthly injections | 24 (47%) |  |  |  |
| - Six-monthly injections | 3 (6%) |  |  |  |
| - Intermittent | 3 (6%) |  |  |  |
| - Tablets | 3 (6%) |  |  |  |
| Radiation types |  |  |  |  |
| - External beam | 22 (43%) |  |  |  |
| - Brachytherapy | 2 (4%) |  |  |  |

## Table S2. Medical comorbidities details

| **Condition** | **Number endorsing ‘Yes’** | **Number endorsing ‘No’** |
| --- | --- | --- |
| Heart condition | 8 | 42 |
| Diabetes | 8 | 43 |
| High blood pressure | 23 | 27 |
| Condition affects the brain (e.g., encephalitis, meningitis, epilepsy, dementia, Parkinson's,etc.?) | 0 | 51 |
| Head trauma* | 4 | 47 |
| Seizures or fits (where you have blacked out) | 0 | 51 |

*The question asked was “Have you ever been knocked unconscious for more than 5 minutes?”

## Table S3. Covariate Analysis with *p-*values

|  | **Occupational outcomes (*p*-values)*** | | |
| --- | --- | --- | --- |
| ***Demographic*** | *(1) Changes to your ability to work?* | *(2) Ability to perform work duties at the same level?* | *(3) Decreased work hours?* |
| Age (*n* = 51) | .401 | .095 | .661 |
| Relationship | .245 | .056 | .437 |
| - Partnered |  |  |  |
| - Not partnered |  |  |  |
| Education level | .598 | .494 | .098 |
| - Did not complete Year 12 |  |  |  |
| - Year 12/ Vocational training |  |  |  |
| - Undergraduate/postgraduate |  |  |  |
| Current employment status | .769 | .776 | 1.00 |
| - Full-time/part-time/looking for employment/self-employed/carer |  |  |  |
| - Retired |  |  |  |
| Employment type | .283 | .785 | .471 |
| - Clerical and sales/service/agricultural/ machines trade/ structural work e.g., construction |  |  |  |
| - Professional, technical and managerial occupations |  |  |  |
| - Other |  |  |  |
| No. of children (n=44) | .806 | .484 | .721 |
| Children living at home | .374 | .547 | .512 |
| - Yes |  |  |  |
| - No |  |  |  |
| Country of birth | .539 | .570 | .545 |
| - Aus/NZ |  |  |  |
| - Other: US/UK/Europe/Asia/South America/ Africa |  |  |  |
| Location of residence | .206 | 1.00 | .746 |
| - Metropolitan |  |  |  |
| - Regional |  |  |  |
| Languages spoken other than English | .251 | .06 | .114 |
| - No |  |  |  |
| - Yes |  |  |  |
| ***Prostate cancer details*** |  |  |  |
| Stage | .574 | .775 | .565 |
| - Early/Regional |  |  |  |
| - Metastatic |  |  |  |
| Time since diagnosis in months (n=50) | .548 | .789 | .603 |
| Cancer treatments |  |  |  |
| - Watchful waiting/active surveillance | .136 | .238 | .237 |
| - Surgery | .768 | .557 | .769 |
| - Hormone therapy (e.g., ADT) | **.007**** | .110 | **.049**** |
| - Radiation therapy | .085 | .775 | .153 |
| - Chemotherapy | .035 | .223 | 1.00 |
| - Immunotherapy | - | - | - |
| ***Medical details*** |  |  |  |
| Number of noncancer medications (n=42) | .721 | .571 | .719 |
| No of comorbidities* (n=50) | **.009**** | .375 | .515 |
| Mental health diagnosis (n=51) | 1.00 | .741 | .297 |

*Categorical and continuous variables analysed using two-tailed Fisher’s exact tests and one-way Analysis of Variance respectively.

** *p*<.05

## S5: SPSS output of logistic regression analysis using PCI20 as a predictor of occupational outcomes

### (1) Changes to your ability to work?


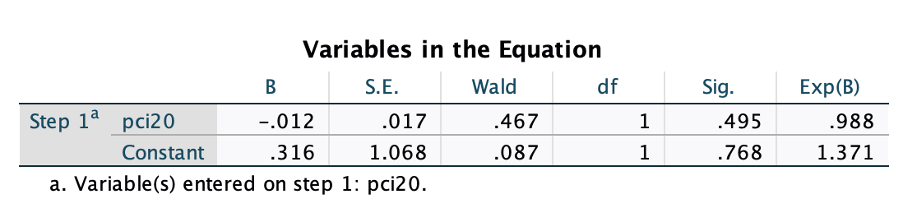


With covariates:


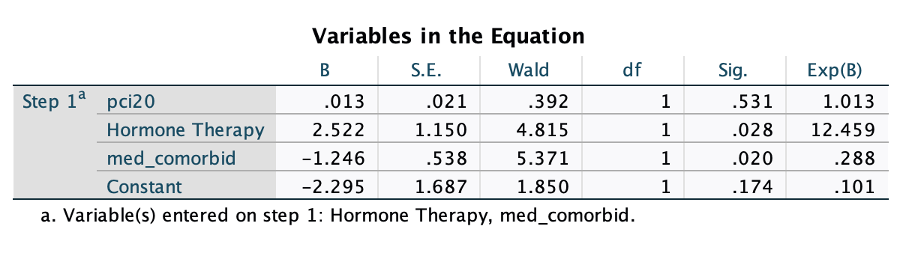


PCI20 using Fardell et al. (2022) cut off for low cognitive functions:


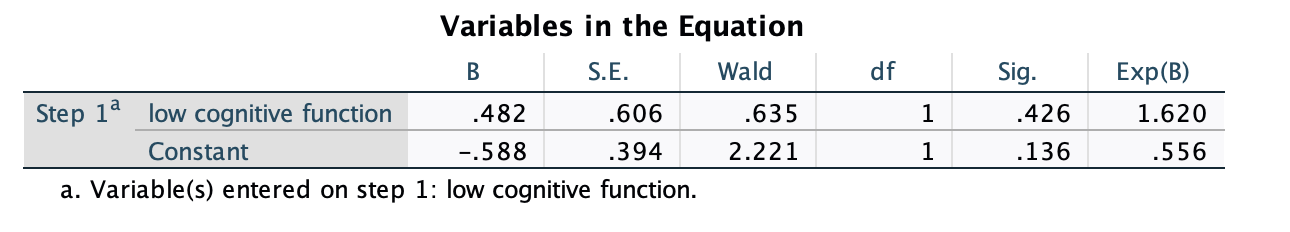


With covariates:


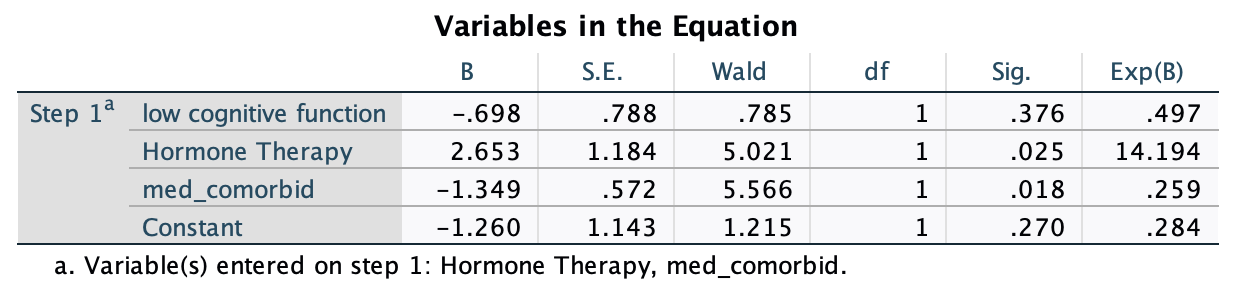


### (2) Ability to perform work duties at the same level?


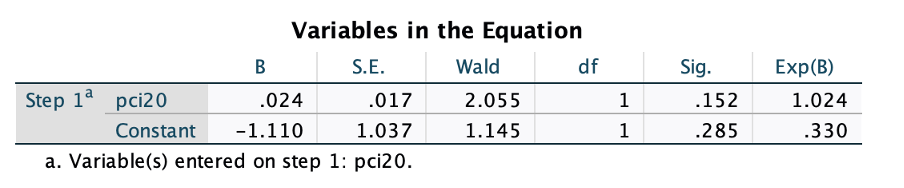


PCI20 using Fardell et al. (2022) cut off for low cognitive functions:


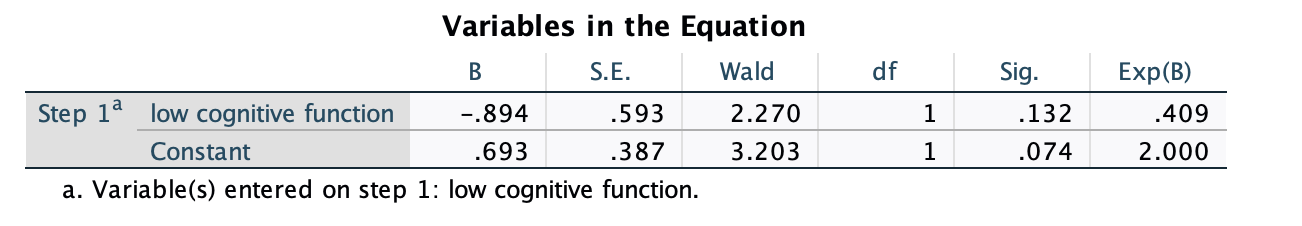


### (3) Decreased work hours?


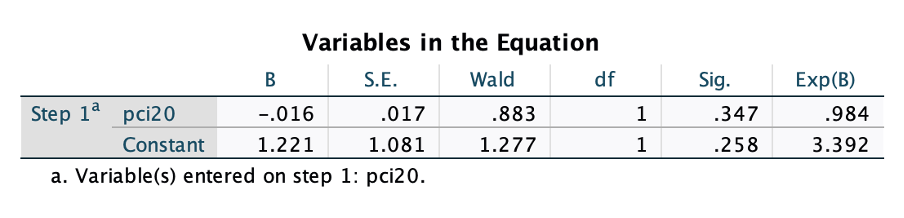


With covariates:


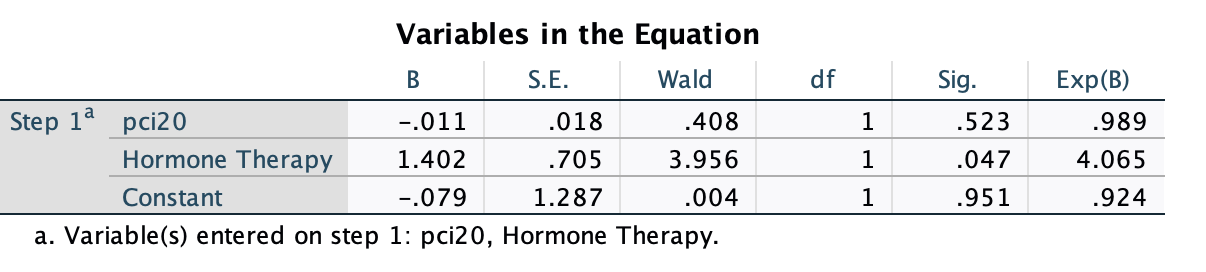


PCI20 using Fardell et al. (2022) cut off for low cognitive functions:


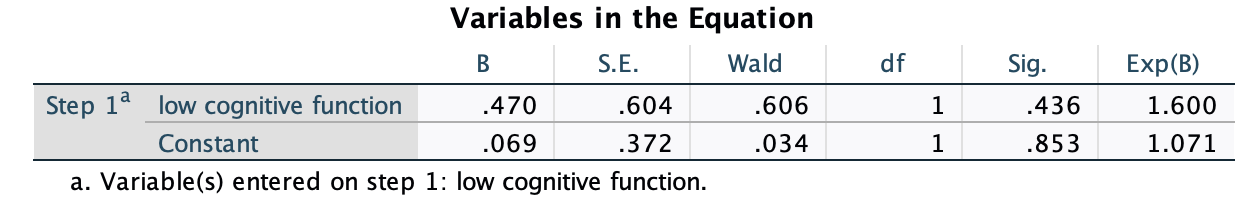


With covariates:


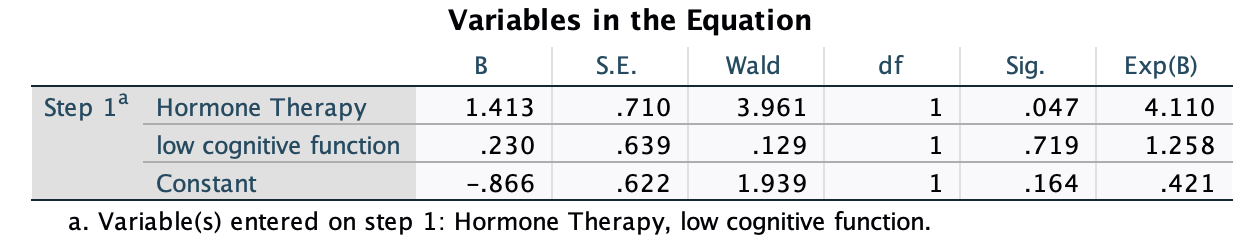


## Table S6. PCI20 Means and Standard Deviations for Occupational Outcomes

|  | *(1) Changes to your ability to work?* | | (2) Ability to perform work duties at the same level? | | (3) Decreased work hours? | |
| --- | --- | --- | --- | --- | --- | --- |
|  | No | Yes | No | Yes | No | Yes |
| Mean | 61.7 | 58.4 | 55.4 | 62.7 | 62.4 | 57.6 |
| SD | 18.3 | 15.6 | 18.1 | 17 | 17.3 | 18.1 |

## S7: SPSS output of logistic regression analysis using EORTC-QLQ CF as a predictor of occupational outcomes

### (1) Changes to your ability to work?


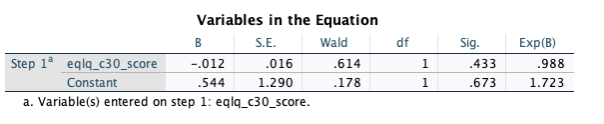


With covariates:


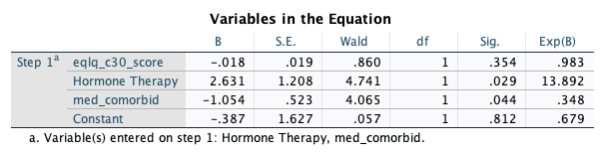


PCI20 using Fardell et al. (2022) cut off for low cognitive functions:


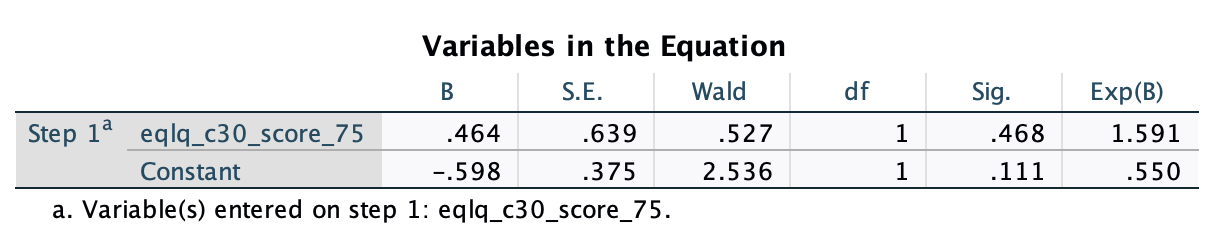


With covariates:


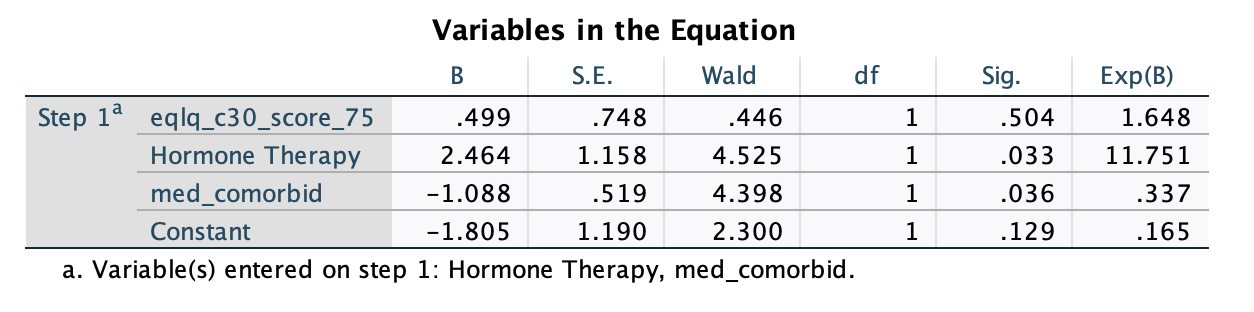


### (2) Ability to perform work duties at the same level?


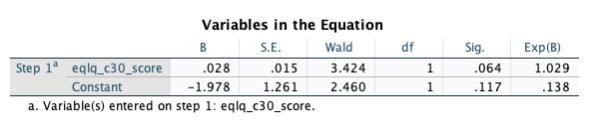


PCI20 using Fardell et al. (2022) cut off for low cognitive functions:


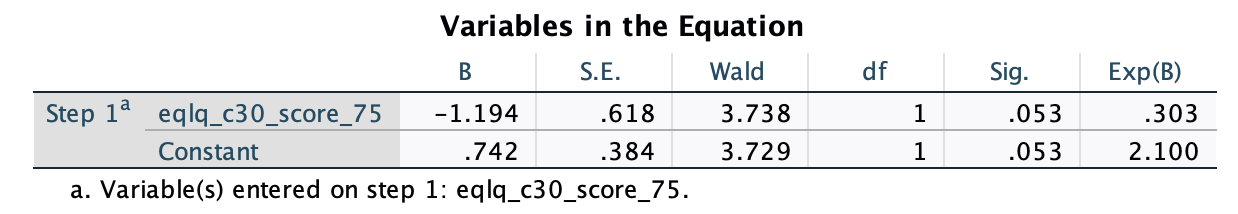


### (3) Decreased work hours?


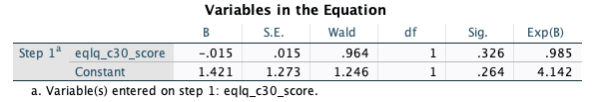


With covariates:


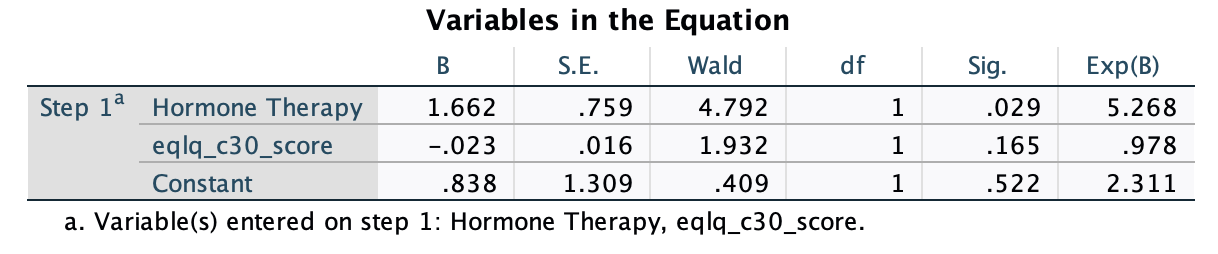


PCI20 using Fardell et al. (2022) cut off for low cognitive functions:


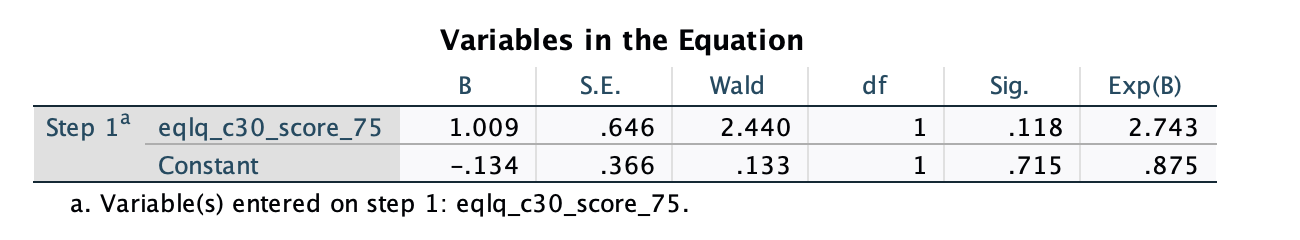


With covariates:


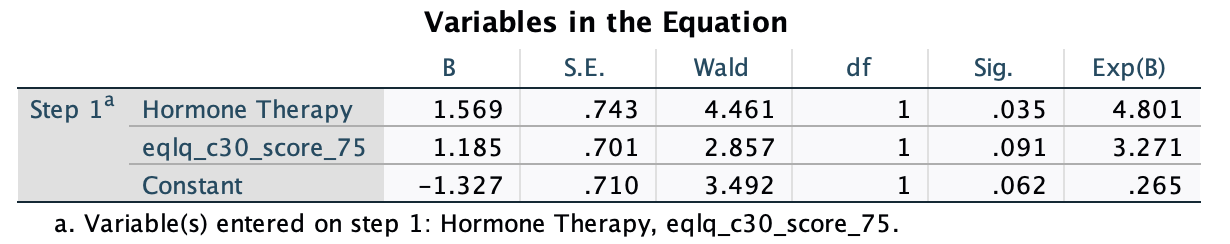


## Table S8. EORTC-CF Means and Standard Deviations for Occupational Outcomes

|  | *(1) Changes to your ability to work?* | | (2) Ability to perform work duties at the same level? | | (3) Decreased work hours? | |
| --- | --- | --- | --- | --- | --- | --- |
|  | No | Yes | No | Yes | No | Yes |
| Mean | 83.9 | 78.1 | 73.8 | 85.1 | 84.1 | 78.2 |
| SD | 20.2 | 18.5 | 22.7 | 17.8 | 18.6 | 22 |
